# Maternal hypothyroxinemia in the first trimester of gestation and association with obstetric and neonatal outcomes and iron deficiency: a prospective Brazilian study

**DOI:** 10.20945/2359-3997000000043

**Published:** 2018-05-07

**Authors:** Pedro Weslley Rosario, Luis Fernando Faria Oliveira, Maria Regina Calsolari

**Affiliations:** 1 Santa Casa de Misericórdia de Belo Horizonte Santa Casa de Belo Horizonte Programa de Pós-Graduação e Serviço de Endocrinologia Belo Horizonte MG Brasil Programa de Pós-Graduação e Serviço de Endocrinologia, Santa Casa de Belo Horizonte, Belo Horizonte, MG, Brasil; 2 Universidade de Itaúna Fundação Universidade de Itaúna Itaúna MG Brasil Fundação Universidade de Itaúna, Itaúna, MG, Brasil

**Keywords:** Pregnancy, first trimester, hypothyroxinemia, iron deficiency, obstetric and neonatal outcomes

## Abstract

**Objective:**

To evaluate the association of isolated hypothyroxinemia in the first trimester with obstetric and neonatal outcomes and iron deficiency.

**Subjects and methods:**

The study was prospective. Women who had become pregnant spontaneously were initially selected. Next, anti-thyroid peroxidase antibodies (TPOAb), free T4 (FT4), total T4 (TT4), TSH, and ferritin were measured. TPOAb-positive women were excluded. The final sample consisted of 596 women with serum TSH between 0.1 and 2.5 mIU/l. Hypothyroxinemia was defined as FT4 < 0.86 ng/dL and < 0.92 ng/dL, corresponding to the 5^th^ and 10^th^ percentiles, respectively, and TT4 < 7.8 ng/dL. None of the pregnant women was treated with levothyroxine until the end of pregnancy.

**Results:**

The women ranged in age from 18 to 36 years, with a median gestation of 9 weeks. T4 levels were not correlated with BMI or maternal TSH. Isolated hypothyroxinemia was observed in 4.3% (FT4 < 0.86 ng/dL), 9% (FT4 < 0.92 ng/dL), and 7% (TT4 < 7.8 ng/dL) of the pregnant women. The frequencies of obstetric and neonatal outcomes were similar in women with versus without hypothyroxinemia. In women without iron deficiency, 8.4%, 3.9%, and 6.5% had FT4 < 0.92 ng/dl, FT4 < 0.86 ng/dL and TT4 < 7.8 ng/dL, respectively. These frequencies of hypothyroxinemia were significantly higher among women with iron deficiency (20.7%, 14.8% and 17.2%, respectively).

**Conclusions:**

This prospective Brazilian study found no association between isolated hypothyroxinemia in the first trimester of gestation and obstetric or neonatal outcomes, but an association was demonstrated with iron deficiency.

## INTRODUCTION

When screening for thyroid dysfunction in pregnancy is indicated, it should be performed in the first prenatal visit, which usually occurs in the first trimester. Possible consequences of subclinical hypothyroidism and isolated maternal hypothyroxinemia also appear to be more worrisome when they occur in the first trimester of gestation, a period when the fetus still does not produce thyroid hormone and depends on maternal thyroxine (T4) (1). It is therefore more important to know the impact of these conditions when diagnosed in the early stages of pregnancy (1,2).

Isolated hypothyroxinemia is diagnosed in the presence of serum TSH within the normal range associated with T4 concentrations below the 2.5^th^, 5^th^ or 10^th^ percentile (2–4). Regarding TSH, except when the assay- and population-specific references values are available, the range of 0.1 to 2.5 mIU/l has been accepted for the definition of maternal euthyroidism in the first trimester (2,3,5–7). It is worth noting that this range does also seem to be adequate for our population (8). Iodine deficiency appears to the most important factor in the etiology of isolated hypothyroxinemia, but other factors have also been implicated, such as environmental disruptors, obesity, and iron deficiency (4). No consensus exists regarding the consequences and need for levothyroxine (L-T4) replacement therapy in isolated hypothyroxinemia (4). The American College of Obstetricians and Gynecologists (ACOG) does not consider this condition in its guidelines (9). The American Thyroid Association (ATA) does not recommend its treatment (3), while the Endocrine Society proposes partial replacement therapy (5). According to the European Thyroid Association (ETA), treatment can be considered when hypothyroxinemia is detected in the first trimester, but not when it is detected in the second or third trimester (2). A large recent study found no benefit of L-T4 replacement therapy in isolated hypothyroxinemia, but one limitation of that study is that this therapy was generally initiated only in the 18^th^ week of gestation (10,11).

In view of the lack of consensus regarding the management of isolated hypothyroxinemia, notably when detected in the first trimester, the objective of this study was to evaluate the association of this condition with obstetric and neonatal outcomes. The association with iron deficiency was also explored. To our knowledge, no Brazilian study has so far evaluated these associations.

## SUBJECTS AND METHODS

The study was prospective and was approved by the local Research Ethics Committee (8).

### Selection

The population studied was from the metropolitan region of Belo Horizonte (Minas Gerais, Brazil). One thousand and eighty two pregnant women who underwent prenatal exams at a clinical analysis laboratory and who had become pregnant spontaneously were initially interviewed and examined (8). Women who met the clinical criteria shown in Table 1 (n = 748) were first selected. Next, anti-thyroid peroxidase antibodies (TPOAb), free T4 (FT4), total T4 (TT4), TSH, and ferritin were measured. TPOAb-positive pregnant women (n = 38) were excluded. Women with hyperemesis gravidarum, twin pregnancy or trophoblastic disease (n = 50) were also excluded. The sample consisted of 660 women and 596 with serum TSH between 0.1 and 2.5 mIU/l (2,3,5-7,8) were selected.

**Table 1:** Inclusion criteria

Clinical criteria
Absence of thyroid disease, no current or previous treatment with antithyroid drugs or L-T4, no history of ^131^I therapy or thyroidectomy
No use of potentially interfering medications such as dopaminergic agonists or antagonists, neuroleptics, corticosteroids, estrogen, amiodarone, interferon, lithium, anticonvulsants, metformin, octreotide; or recent (in the past 8 weeks) exposure to iodinated contrast agents
No history of head and neck external radiotherapy
Absence of type 1 diabetes or other autoimmune diseases
No family history of thyroid disease
Absence of goiter or any palpable thyroid anomaly
Absence of ophthalmopathy
Complementary criteria: ≤ 12 weeks gestation

### Protocol

At the time of the study, and still today, T4 measurement is not necessary in women with TSH ≤ 2.5 mIU/L without TPOAb. There was also, and continues to be, no consensus recommendation regarding the treatment of hypothyroxinemia. Hence, in order to avoid interference with the results, the protocol was approved foreseeing the non-repetition of thyroid function tests during pregnancy and was thus executed. None of the pregnant women was treated with L-T4 until the end of pregnancy. Regarding iron supplementation, the women only received the recommended daily doses in all pregnant women without additional supplementation in those with iron deficiency.

### Outcomes

Obstetric and neonatal outcomes were compared between women with FT4 < 0.86 ng/dL and < 0.92 ng/dL and those with FT4 ≥ 0.92 ng/dL. These values correspond, respectively, to the 5^th^ and 10^th^ percentiles of FT4 concentrations obtained for the initially selected 660 TPOAb-negative women (8). We also compared women with TT4 < 7.8 ng/dL and ≥ 7.8 ng/dL. This value is provided by the assay as the lower limit of normal for the first trimester of gestation and corresponds exactly to 1.5 times the lower limit of the reference range for non-pregnant healthy adults (12). The outcomes (reported in Table 2) were predefined before the start of the study based on those used in the largest series that evaluated the association between hypothyroxinemia and pregnancy outcomes (13). The definitions of hypothyroxinemia were also pre-established.

**Table 2:** Obstetric outcomes

Obstetric outcomes	FT4 (ng/dL)			TT4 (μg/dL)	
	< 0.86* (n = 26)*	< 0.92** (n = 54)**	≥ 0.92* ** (n = 542)* **	< 7.8*** (n = 42)***	≥ 7.8*** (n = 554)***
Hypertensive disease of pregnancy	2 (7.7%)*	5 (9.2%)**	47 (8.6%)* **	4 (9.5%)***	50 (9%)***
Gestational mellitus diabetes	3 (11.5%)*	6 (11.1%)**	56 (10.3%)* **	5 (12%)***	60 (10.8%)***
Placental abruption	0*	0**	2 (0.3%)* **	0***	2 (0.3%)***
Delivery before 34 weeks of gestation	0*	1 (1.8%)**	5 (0.9%)* **	1 (2.4%)***	5 (0.9%)***
Delivery before 37 weeks of gestation	1 (3.8%)*	2 (3.7%)**	15 (2.7%)* **	2 (4.7%)***	16 (2.8%)***
Fetal loss (after initial evaluation)	0*	2 (3.7%)**	16 (2.9%)* **	2 (4.7%)***	16 (2.8%)***

FT4: free T4; TT4: total T4.

* FT4 < 0.86 ng/dL versus ≥ 0.92 ng/dL: p ≥ 0.5 in all comparisons.

** FT4 < 0.92 ng/dL versus ≥ 0.92 ng/dL: p ≥ 0.65 in all comparisons.

*** TT4 < 7.8 mg/dL versus TT4 ≥ 7.8 ng/dL: p ≥ 0.35 in all comparisons.

### Laboratory

Iron deficiency was defined as a ferritin concentration < 15 ng/mL (14). TSH, FT4, TT4 and TPOAb were measured with a chemiluminescent assay (Diagnostic Products Corporation, Los Angeles, CA). Serum samples were obtained from the women in the morning (at about 8 a.m.) after an 8- to 10-h fast (8).

### Statistical

The Spearman or Pearson correlation test was used to analyze the correlation between T4 and body mass index (BMI) and TSH. The t-test or Mann-Whitney test and Fisher's exact test or the χ^2^ test were used for comparison between groups. A P value < 0.05 was considered statistically significant.

## RESULTS

The characteristics of the 596 patients are shown in Table 3.

**Table 3:** Characteristics of the 596 patients

Age [range, median (years)]	18-36 (27)
Body mass index [range, median (kg/m^2^)]	18-30 (22.6)
Gestational age [range, median (weeks)]	7-12 (9)
Primigravidae	312 (52.3%)
TSH [range, median (mIU/L)]	0.1-2.5 (0.98)
Hypothyroxinemia	Free T4 < 0.86 ng/dL: 26 (4.3%) Free T4 < 0.92 ng/dL: 54 (9%) Total T4 < 7.8 ng/dL: 42 (7%)
Iron deficiency	29 (4.8%)

FT4 levels were not correlated with BMI (p = 0.6) or maternal TSH (p = 0.1). TT4 levels were also not correlated with BMI (p = 0.7) or maternal TSH (p = 0.15).

Tables 2 and 4 show the obstetric and neonatal outcomes, respectively, according to maternal T4 concentrations in the first trimester of gestation. The groups were similar in terms of age, BMI and maternal TSH levels. No difference in the frequencies of obstetric or neonatal outcomes was observed, regardless of the definition of isolated hypothyroxinemia.

**Table 4:** Neonatal outcomes

Neonatal outcomes	FT4 (ng/dL)			TT4 (μg/dL)	
	< 0.86* (n = 26)*	< 0.92** (n = 54)**	≥ 0.92* ** (n = 542)* **	< 7.8*** (n = 42)***	≥ 7.8*** (n = 554)***
Birth weight < 2,500 g	1 (3.8%)*	2 (3.7%)**	32 (5.9%)* **	2 (4.7%)***	33 (5.9%)***
Birth weight < 1,500 g	0*	1 (1.8%)**	8 (1.4%)* **	1 (2.3%)***	8 (1.6%)***
Need for intensive therapy	0*	1 (1.8%)**	11 (2%)* **	1 (2.3%)***	11 (2%)***
Need for mechanical ventilation > 24 h	0*	1 (1.8%)**	11 (2%)* **	1 (2.3%)***	11 (2%)***
Necrotizing enterocolitisᵃ	0*	0**	1 (0.2%)* **	0***	1 (0.2%)***
Intraventricular hemorrhage grade 3 or 4	0*	0**	0* **	0***	0
Major congenital malformations	0*	1 (1.8%)**	5 (0.9%)* **	0***	6 (1%)***
Neonatal death (< 28 days)	0*	0**	3 (0.5%)* **	0***	3 (0.5%)***

FT4: free T4; TT4: total T4.

a With need for surgery.

* FT4 < 0.86 ng/dL versus ≥ 0.92 ng/dL: p ≥ 0.8 in all comparisons.

** FT4 < 0.92 ng/dL versus ≥ 0.92 ng/dL: p ≥ 0.45 in all comparisons.

*** TT4 < 7.8 ng/dL versus TT4 ≥ 7.8 ng/dL: p ≥ 0.5 in all comparisons.

Iron deficiency was diagnosed in 29 (4.8%) pregnant women. This frequency was 4.2% in women with FT4 ≥ 0.92 ng/dL. The frequencies of iron deficiency were significantly higher in women with FT4 < 0.86 ng/dL and < 0.92 ng/dL (hypothyroxinemia), 15.4% (p = 0.03) and 11.1% (p = 0.04), respectively. Women with TT4 < 7.8 ng/dL (hypothyroxinemia) also exhibited a higher frequency of iron deficiency than women with TT4 ≥ 7.8 ng/dL (12% versus 4.3%, p = 0.045). In women without iron deficiency, 8.4%, 3.9% and 6.5% had FT4 < 0.92 ng/dL, FT4 < 0.86 ng/dL and TT4 < 7.8 ng/dL respectively. These frequencies of hypothyroxinemia were significantly higher among women with iron deficiency, 20.7% (p = 0.038), 14.8% (p = 0.032) and 17.2% (p = 0.045), respectively.

## DISCUSSION

Some characteristics of the present study should be highlighted. This was a prospective study. Hypothyroxinemia was defined using three different criteria of T4 concentration. Regarding TSH, a cutoff of 2.5 mIU/l was used since it is still the most accepted value for the first trimester of gestation (2,3,5–7) and is compatible with the normal range for our population (8). In addition, an overestimated TSH cutoff may compromise the conclusions because of the inclusion of women with subclinical hypothyroidism. Women with TPOAb were excluded to rule out the possible interference of thyroid autoimmunity with the outcomes. The women were evaluated in the first trimester since eventual repercussions of isolated hypothyroxinemia would be greater during this period (1,2,11) and treatment is particularly considered in this stage of pregnancy (2). None of the women was treated with L-T4 during pregnancy. Finally, few studies have investigated the relationship between hypothyroxinemia and iron deficiency. To our knowledge, this is the first Brazilian study analyzing the association of hypothyroxinemia with obstetric and neonatal outcomes and iron deficiency.

Regarding the association of hypothyroxinemia with iron deficiency, our results confirm the data of a Chinese observational study, which also evaluated women in the first trimester of gestation from an iodine-sufficient area (15). Since it would be more than a coincidence that iron and iodine deficiencies are the consequence of broad nutritional deficiency or that isolated hypothyroxinemia (in the absence of hypothyroidism) causes iron deficiency, iron deficiency is more likely to contribute to hypothyroxinemia (15). It is possible that iodine sufficiency influences the consequence of iron deficiency in terms of thyroid dysfunction, whether it is predominantly hypothyroxinemia (without TSH elevation) in an iodine-sufficient population (15) like ours, or subclinical hypothyroidism in an iodine-deficient population (16). However, further clinical studies involving pregnant women are necessary to consistently confirm this association.

We found no association between isolated hypothyroxinemia in the first trimester of gestation and obstetric or neonatal outcomes. Casey and cols., comparing 16,011 women with normal TSH and T4 and 233 with hypothyroxinemia before 20 weeks of gestation, also observed no association between this alteration and any of the obstetric or neonatal outcomes analyzed (13). Cleary-Goldman and cols. evaluated 10,021 pregnant women with normal TSH and T4 and 232 with hypothyroxinemia (17). In the first trimester, hypothyroxinemia was associated with preterm labor (adjusted odds ratio [aOR] 1.62; 95% confidence interval [CI] 1.00-2.62) and macrosomia (aOR 1.97; 95% CI 1.37-2.83) (17). In the second trimester, it was associated with gestational diabetes (aOR 1.7; 95% CI 1.02-2.84) (17). Finally, a third study involving more than 5,000 women revealed an association of hypothyroxinemia with preterm labor (18), but not with hypertensive disease in pregnancy (19). A meta-analysis that included these and other smaller studies found only a statistically significant increased risk of placental abruption (OR 2.3; 95% CI: 1.1–4.8) compared to euthyroid controls (20). Taken together, these findings show that the association of hypothyroxinemia with obstetric and neonatal outcomes is still inconsistent (4). In fact, there is no reproducibility of the results in the different studies and no cause-effect relationship could be established.

We recognize some limitations of the study. First, the number of women with hypothyroxinemia may have been insufficient to obtain statistical significance of small differences compared to euthyroid women. Second, since this was not an intervention study, the objective was limited to demonstrating the association between iron deficiency and hypothyroxinemia, but we did not evaluate the effect of iron supplementation on T4 concentrations. Finally, we analyzed obstetric and neonatal outcomes but not parameters of fetal brain development, which could also be compromised by isolated hypothyroxinemia (3). Although an association between hypothyroxinemia at the beginning of pregnancy and some impairment of fetal brain development has been reported in some series (3,20), two randomized studies found no beneficial effect of L-T4 therapy on this aspect (10,21).

In conclusion, this prospective Brazilian study found no association of isolated hypothyroxinemia in the first trimester of gestation with obstetric or neonatal outcomes. Despite the need for confirmation in larger series, this result weakens the concern regarding serum T4 in pregnant women with normal TSH, i.e., with isolated hypothyroxinemia. In contrast, an association was demonstrated between hypothyroxinemia and iron deficiency. This finding suggests the importance of iron and possible repercussions of its deficiency on thyroid function and the need for intervention studies evaluating the effects of iron supplementation on thyroid status in pregnant women.
